# First-in-Human Study of ^18^F-Labeled PET Tracer for Glutamate AMPA Receptor [^18^F]K-40: A Derivative of [^11^C]K-2

**DOI:** 10.2967/jnumed.124.269405

**Published:** 2025-06

**Authors:** Sadamitsu Ichijo, Tetsu Arisawa, Mai Hatano, Waki Nakajima, Tomoyuki Miyazaki, Tsuyoshi Eiro, Yuuki Takada, Ryunosuke Iai, Akane Sano, Masaki Sonoda, Yutaro Takayama, Yuichi Kimura, Takuya Takahashi

**Affiliations:** 1 Department of Physiology, Yokohama City University Graduate School of Medicine, Yokohama, Japan;; 2 Yokohama City University Graduate School of Medicine, Radioisotope Research Center, Yokohama, Japan;; 3 Center for Promotion of Research and Industry–Academic Collaboration, Yokohama City University, Yokohama, Japan;; 4 Department of Psychiatry, Yokohama City University Graduate School of Medicine, Yokohama, Japan;; 5 Department of Neurosurgery, Yokohama City University Graduate School of Medicine, Yokohama, Japan; and; 6 Faculty of Informatics, Cyber Informatics Institute, Kindai University, Higashi-Osaka, Japan

**Keywords:** PET, AMPA receptor, neuroimaging, first-in-human study

## Abstract

Although the alteration of glutamate α-amino-3-hydroxy-5-methyl-4-isoxazole propionic acid receptor (AMPAR) distribution is believed to underlie physiologic and pathologic neuronal function, there has been no modality to evaluate AMPARs in a living human. [^11^C]K-2, the PET tracer we previously developed, is the first and only technology, to the best of our knowledge, to visualize AMPAR densities in the living human brain. Despite its favorable kinetics as a PET tracer, the short half-life of ^11^C limits the potential of [^11^C]K-2. We recently developed an ^18^F-labeled PET tracer, [^18^F]K-40, which demonstrated AMPAR-specific binding properties and brain distribution similar to that of [^11^C]K-2 in preclinical studies. The purpose of this first-in-human study is to evaluate the properties of [^18^F]K-40 in humans and to compare the kinetics and PET images of [^18^F]K-40 with those of [^11^C]K-2. **Methods**: Five healthy volunteers were enrolled and underwent dynamic PET imaging using [^18^F]K-40 and [^11^C]K-2. The nondisplaceable binding potential (BP_ND_) with white matter as the reference was calculated by Logan graphical analysis using tissue time–activity curves (TACs), and the total distribution volume of [^18^F]K-40 was calculated using plasma TACs. The intraindividual correlation between BP_ND_ values obtained for [^18^F]K-40 and [^11^C]K-2 was examined. To optimize the time window for PET scanning, BP_ND_ and SUV ratio were evaluated. **Results**: The tissue TACs of [^18^F]K-40 showed curves similar to those of [^11^C]K-2. Logan graphical analysis using plasma TACs revealed reversible binding of [^18^F]K-40. The BP_ND_ obtained with [^18^F]K-40 and [^11^C]K-2 significantly correlated in each corresponding region and showed very good correlation, which indicated that K-40, as observed with K-2, can provide PET images that reflect the amount of AMPARs. A good linear relationship was observed between BP_ND_ and the summation image of SUV ratios between 40 and 50 min after radiotracer injection. **Conclusion**: [^18^F]K-40, as with [^11^C]K-2, has favorable binding properties as an AMPAR PET tracer. Thus, [^18^F]K-40 could characterize AMPAR distribution in pathophysiologic conditions of the brain and facilitate the development of novel diagnostics of neuropsychiatric disorders.

## Visual Abstract

**Figure:**
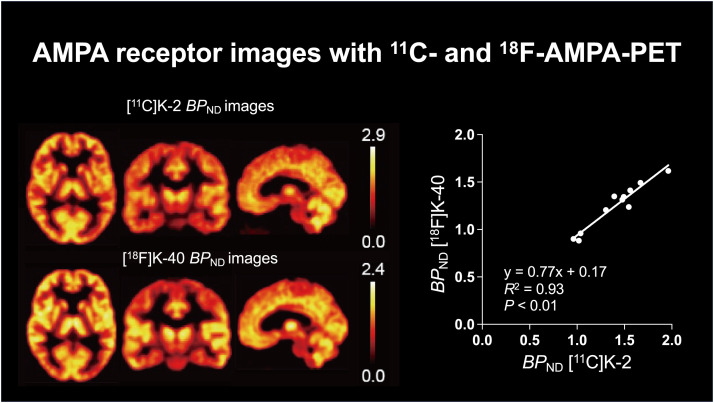

Glutamate α-amino-3-hydroxy-5-methyl-4-isoxazole propionic acid receptors (AMPARs) are a subtype of glutamate receptors and play a pivotal role in neuronal excitatory synaptic transmission (1–8). Many animal studies have suggested that changes in the number of synaptic AMPARs underlie physiologic function, including learning and memory (1–8). Furthermore, aberrant distribution and malfunction of AMPARs are thought to underlie neuropsychiatric disorders, such as epilepsy, dementia, schizophrenia (9–11). Despite numerous animal and postmortem studies suggesting the potential of AMPARs as diagnostic and therapeutic targets for neuropsychiatric disorders, the clinical translation has been limited. This could be attributed to the lack of a modality for AMPAR quantification in the living human brain, as the trafficking of AMPARs is activity-dependent, which is difficult to characterize with postmortem brain studies.

PET is a nuclear medicine imaging modality widely used in clinical diagnosis. Despite the development of many AMPAR-targeted PET probes, the development of a practical PET probe with balanced functions of brain uptake, AMPAR-binding ability, and blood clearance poses a challenge (12–17).

A PET probe, [^11^C]K-2, that successfully visualizes cell-surface AMPARs in the living human brain (18–20), was used to quantify AMPAR densities in patients with 4 major psychiatric disorders, including schizophrenia, bipolar disorder, depression, and autism spectrum disorder (21), and demonstrated strong significant correlations between AMPAR density in specific brain areas and the symptomatology scores for each psychiatric disorder (21). Some brain areas showed significant differences in AMPAR density in patients with each psychiatric disorder compared with healthy subjects (21). Some of these overlapped across diseases, indicating that these areas are commonly affected regions of the brain throughout psychiatric disorders (21).

In patients with focal epilepsy, the amplitude of abnormal γ-activity was positively correlated with regional AMPAR density. Meanwhile, AMPAR densities in some regions were significantly lower compared with healthy subjects, suggesting that Hebbian plasticity and homeostatic scaling regulate epileptic brain function (22).

These results indicate that [^11^C]K-2 has sufficient potential to elucidate the physiologic and pathologic alterations of AMPAR density in the human brain. However, the short half-life (20 min) of the radioisotope ^11^C necessitates immediate postproduction administration of [^11^C]K-2 that warrants an on-site cyclotron. Therefore, the development of a new tracer labeled with a radioisotope with a longer half-life is desirable.

The radioisotope ^18^F has a longer half-life (∼110 min), and recently, we attempted the development of an ^18^F-labeled AMPAR PET probe.

For screening of the ^18^F-labeled AMPAR PET probe, the candidate compounds were synthesized on the basis of the structures of [^11^C]K-2, which has been previously established as an AMPAR PET tracer. The AMPAR PET suitability of the candidate compounds was evaluated by small-animal PET imaging and comparison with K-2. For the candidate compounds that provided favorable images, specificity was evaluated using rats in which the AMPAR was knocked down by short hairpin RNA technology, and K-40 was ultimately identified as the most promising compound (23).

In this clinical study, we performed both [^11^C]K-2 and [^18^F]K-40 PET scans on the same subjects to investigate the applicability of [^18^F]K-40 as an AMPAR PET tracer.

## MATERIALS AND METHODS

### Ethics Statement

The clinical study protocol was approved by Yokohama City University Human Investigation Committee in accordance with the ethical guidelines for medical and health research involving human subjects issued by the Japan Ministry of Health, Labor, and Welfare, and the study was registered (ID: jRCTs031220226). The date of the first and last treatment was September 26, 2022, and December 5, 2022, respectively. All subjects provided written informed consent.

### Radiosynthesis of [^18^F]K-40 and [^11^C]K-2

[^18^F]K-40 and [^11^C]K-2 were synthesized as described previously (20,23). The specifications for [^18^F]K-40 and [^11^C]K-2 injection were set as described in Tables 1 and 2. All materials were tested for each production run. As an exception, the sterility test results were confirmed 2 wk after production and injection.

**TABLE 1.** Specification of [^18^F]K-40

Test	Specification
1. Quantity	
1.1 Volume of one batch	18–22 mL
1.2 Radioactivity	NLT 1.85 GBq
1.3 K-40 concentration	NMT 10 μg/mL
1.4 Specific radioactivity	NLT 37 GBq/μmol on EOS
1.5 Half-life	NLT 105 min; NMT 115 min
2. Property	
2.1 Appearance	Colorless to light yellow solution
2.2 Particle	Free of visible particulate matter
3. Bacterial endotoxin	NMT 150 EU/whole solution
4. Sterility	No growth
5. pH	NLT 5.0; NMT 8.0
6. Qualitative analysis	
6.1 γ-spectrum*	511-keV peak detected
6.2 Radiochemical identity	Radiometric RT ratio of [^18^F]K-40 to K-40 standard: 1.0 ± 0.1
7. Purity	
7.1 Other radioisotope*	No peak except 511 keV and 1,022 keV
7.2 Radiochemical purity	NMT 95%
8. Residual solvent	
8.1 Methanol	NLT 3,000 ppm
8.2 Acetonitrile	NLT 410 ppm
8.3 DMSO	NLT 5,000 ppm
9. Assay of ethanol	NLT 6%; NMT 10%

* All tests are required for every production (6.1 and 7.1 are required once/year).

NLT = not less than; NMT = not more than; EOS = end of synthesis; RT = retention time; DMSO = dimethylsulfoxide.

**TABLE 2.** Specification of [^11^C]K-2

Test	Specification
1. Quantity	
1.1 Volume of one batch	13–17 mL
1.2 Radioactivity	NLT 1.85 GBq
1.3 K-2 concentration	NMT 10 μg/mL
1.4 Specific radioactivity	NLT 37 GBq/μmol on EOS
1.5 Half-life	NLT 19.0 min; NMT 21.0 min
2. Property	
2.1 Appearance	Colorless to light yellow solution
2.2 Particle	Free of visible particulate matter
3. Bacterial endotoxin	NMT 150 EU/whole solution
4. Sterility	No growth
5. pH	NLT 5.0; NMT 8.0
6. Qualitative analysis	
6.1 γ-spectrum*	511-keV peak detected
6.2 Radiochemical identity	Radiometric RT ratio of [^11^C]K-2 to K-2 standard: 1.0 ± 0.1
7. Purity	
7.1 Other radioisotope*	No peak except 511 keV and 1,022 keV
7.2 Radiochemical purity	NMT 95%
8. Residual solvent	
8.1 Acetonitrile	NLT 410 ppm
8.2 DMF	NLT 880 ppm
9. Assay of ethanol	NLT 3%, NMT 6%

* All tests are required for every production (6.1 and 7.1 are required once/year).

NLT = not less than; NMT = not more than; EOS = end of synthesis; RT = retention time; DMF = dimethylformamide.

### In Vitro Binding Assay

Binding affinity assays of K-40_OH_ for AMPARs were performed as previously described (20). In vitro binding selectivity assays were performed by Sekisui Medical to examine the ability of K-40_OH_ to interact with various receptors and channels (off-target binding assay at a concentration of 10 μM; study AL-8801). Details are described in the supplemental materials (available at http://jnm.snmjournals.org).

### Human Subjects

A sample size of 5 was set, and only men were included under ethical consideration. The eligible subjects were individuals aged 20–39 y without any history of neuropsychiatric disorders and had sufficient discriminatory ability to provide consent, according to the MacArthur Competence Assessment Tool for Treatment. No subject had any current mental disorders, based on the criteria in the Structured Clinical Interview for the Diagnostic and Statistical Manual of Mental Disorders, DSM-IV, DSM-5, and ICD10 (24–26). The inclusion and exclusion criteria are provided in the supplemental materials.

A total of 5 healthy subjects met the eligibility criteria and were registered (5 men; age, 25.8 ± 7.5 y). Each subject underwent MRI, PET with [^18^F]K-40, and arterial blood sampling. [^11^C]K-2 PET scanning was performed 2–4 wk after [^18^F]K-40 PET scanning. All tests were performed at Yokohama City University Hospital.

### In Vivo PET and MRI

[^18^F]K-40 and [^11^C]K-2 were synthesized at Yokohama City University Hospital in accordance with the good manufacturing practices ordinance and certified by the Japanese Society of Nuclear Medicine. PET imaging was performed around 2:00 to 4:00 pm using a Discovery MI scanner (GE HealthCare). MRI was performed on a GE Discovery MR750 (GE HealthCare). Details of PET and MRI were described in supplemental materials.

### Measurement of Arterial Contents of Unmetabolized and Metabolized [^18^F]K-40

During the [^18^F]K-40 scans, arterial blood sampling was performed to analyze the unmetabolized and metabolized fractions at 1.5, 3, 7, 12, 20, 30, and 60 min after radiotracer injection. Blood samples were centrifuged at 1,500 *g* for 5 min at 4°C, and the supernatant was collected as the plasma fraction. Subsequently, 0.1% trifluoroacetic acid in a 2:1 (v/v) mixture of acetonitrile and water was added to the plasma, followed by centrifugation at 15,000 *g* for 10 min. To measure the unmetabolized and metabolized [^18^F]K-40 components, the supernatant was filtered off and then analyzed by radio–high-performance liquid chromatography, using a combination of a 1260 Infinity II LC system (Agilent) with a COSMOSIL πNAP packed column (5 μm, 10-mm inner diameter × 250 mm; Nacalai Tesque) and a FlowCountPRO with a bismuth germanate detector flow-scintillation analyzer (Eckert & Ziegler). The mobile phase consisted of 0.1% trifluoroacetic acid in a 55:45 (v/v) mixture of acetonitrile and water (flow rate, 4 mL/min). Finally, the ratio of unmetabolized to metabolized [^18^F]K-40 was calculated.

### PET Imaging Analysis

PET images and T1-weighted MR images were normalized to Montreal Neurological Institute space using the PMOD PNEURO tool version 3.7 (PMOD Technologies) to facilitate use of the N30R83 atlas. The volumes of interest were automatically obtained using the most likely localization of the brain areas encoded in the N30R83 maximum-probability atlas (27). The volume of interest of the white matter, which was the reference region, was obtained using the original script (20). The tissue time–activity curves (tTACs) were then generated for these regions.

### Logan Graphical Analysis (LGA)

LGA was applied to compute nondisplaceable binding potential (BP_ND_) and produce the images (28,29). The BP_ND_ is a quantitative unitless index of receptor density that is defined as the ratio of the amount of available receptor sites to the disassociation rate between an administered radiopharmaceutical and its specific binding sites (30). As LGA is implemented as a linear regression, stable and fast computation is expected and is applicable to any compartment model, such as the 1-tissue–2-compartment or 2-tissue–3-compartment model. If the kinetics of the administered radioligand exhibit reversible behavior, we can compute BP_ND_ using LGA. Therefore, they are commonly used in PET receptor imaging. The tTAC in the reference region, a white matter region, was given to the LGA. The beginning of the linear regression is 20 min after administration. The total distribution volume (*V*_T_) is computed using the metabolite-unadjusted plasma time–activity curve (pTAC) and tTAC (Eq. 1 of (31)) because both the parent and metabolite fractions can enter the brain tissue via the blood–brain barrier.

### Optimal Scan Protocol for SUV Ratio (SUVR) Representing AMPAR Density

We searched an optimal scan protocol to acquire SUVR images that represent the AMPAR density. The acquired dynamic data were interpolated to compute the PET values at any time, and then SUVRs were computed. The scan start time varied from 10 to 70 min after administration, and the frame widths were investigated from 5 to 20 min. These computed SUVR was compared with BP_ND_.

### Statistical Analysis

Statistical analyses were conducted in GraphPad Prism 10 (GraphPad Software), and the data are expressed as mean ± SD, unless indicated otherwise. Correlation coefficients were calculated using Pearson correlation analysis. The results are displayed as *r*^2^ values. Statistical significance was set at a *P* value of less than 0.05. Detailed statistical information for each experiment is provided in the corresponding figure legends.

## RESULTS

### Safety

**FIGURE 1.**
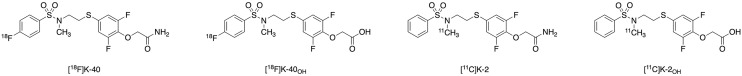
Chemical structures of [^18^F]K-40, [^18^F]K-40_OH_, [^11^C]K-2, and [^11^C]K-2_OH_.

Five healthy subjects underwent a PET scan with [^18^F]K-40 (Fig. 1) and [^11^C]K-2. The mean administration doses of [^18^F]K-40 and [^11^C]K-2 were 186 ± 7 MBq (range, 176–193 MBq) and 378 ± 8 MBq (range, 365–387 MBq), respectively. No significant alterations from baseline biochemistry, hematology, or urine laboratory values were noted, and no significant adverse events were observed during the 7-d observation period after the administration of [^18^F]K-40.

### Plasma Metabolites

Only one metabolite of [^18^F]K-40 was detected in the blood: [^18^F]K-40_OH_ (Supplemental Fig. 1), which was formed by the hydrolysis of the terminal amide group to a carboxyl group, similarly as the previously reported AMPAR PET drug, [^11^C]K-2, is metabolized to [^11^C]K-2_OH_ (Fig. 1) (18,20). The structure of the metabolite K-40_OH_ was identified using the retention time of high-performance liquid chromatography, which matched that of a separately synthesized standard of K-40_OH_ (Supplemental Fig. 1). Chemical structures of [^18^F]K-40, [^18^F]K-40_OH_, [^11^C]K-2, and [^11^C]K-2_OH_ are shown in Figure 1.

### pTAC Analysis

**FIGURE 2.**
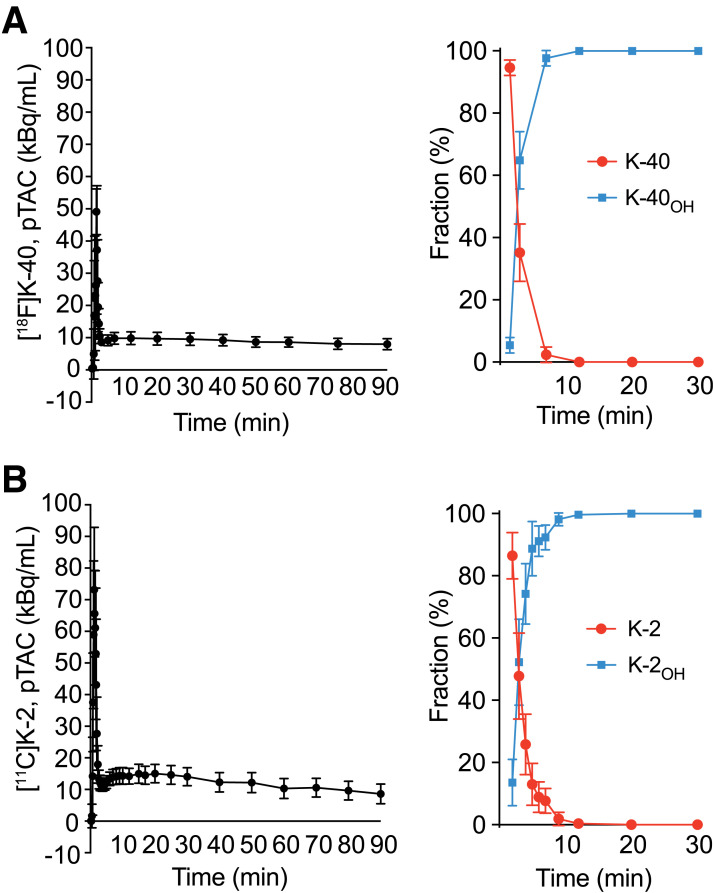
Averaged pTAC (left panels) and metabolic rate (right panels) of [^18^F]K-40 and [^11^C]K-2. Studies for [^18^F]K-40 (*n* = 5) (A). Studies for [^11^C]K-2 (*n* = 6) (B). Data in (B) were obtained from previous study (20). Data are shown as mean ± SD.

Analysis of the radioactivity and metabolites in the plasma obtained from arterial blood samplings after [^18^F]K-40 administration showed that plasma radiation levels increased sharply immediately after administration, quickly decreased to a low level, and decreased gradually thereafter. Figure 2A shows the mean pTAC and the parent and metabolite fractions of K-40, which was metabolized to K-40_OH_ immediately after administration. Half of the K-40 was metabolized 3 min after administration and was almost all completely metabolized 10 min later. Figure 2B shows the mean pTAC and parent and metabolite fractions of [^11^C]K-2, which show similarities in the kinetics of K-40 and K-2 in humans. The detailed plasma time activities of [^11^C]K-2, [^18^F]K-40, and each metabolite are shown in Supplemental Figure 2.

### tTAC Analysis

**FIGURE 3.**
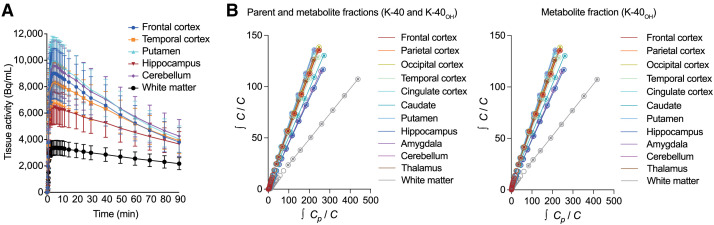
(A) Averaged time–activity curve in brain regions of healthy subjects injected with [^18^F]K-40 (*n* = 5). (B) Typical LGA plots. Estimated amount of K-40 and K-40_OH_ was integrated in left panel, and estimated amount of K-40_OH_ was integrated in right panel. Data are shown as mean ± SD. *C* and *C*_*p*_ are time–activity curves in tissue and arterial plasma. *C*_*p*_ is combined radioactivity of K-40 and K-40_OH_ (B, left) and radioactivity of K-40_OH_ (B, right).

The subject’s brain was divided into several volumes of interest, and Figure 3A shows a graph of the time course of radioactivity in each volume of interest based on dynamic PET imaging data using [^18^F]K-40. This tTAC shows rapid radiotracer uptake in the brain and regional heterogeneities, with the lowest radioactivity in white matter, in which no AMPARs were detected previously (20).

### LGA

LGA was performed using the plasma and tissue radioactivity concentrations obtained from the PET images. Figure 3B shows the typical LGA plots of a representative subject. As K-40 in the blood is rapidly metabolized to K-40_OH_, 2 types of LGA were performed: Figure 3B left, using the radioactivity value in plasma that combines the parent substance (K-40) and metabolite (K-40_OH_), and Figure 3B right, using only the radioactivity of the metabolite. In either case, the LGA showed a good linear relationship in all brain regions, indicating that this PET drug reached a reversible equilibrium state between binding to AMPARs and plasma. Further, these 2 LGA exhibited almost identical slopes, suggesting that [^18^F]K-40_OH_ constitutes AMPAR PET image after the [^18^F]K-40 injection.

### Affinity and Selectivity of K-40_OH_ for AMPARs

Since [^18^F]K-40_OH_ constitutes an AMPAR PET image after the [^18^F]K-40 injection, we focused on K-40_OH_ for further in vitro analysis. To analyze the binding property of K-40_OH_ for AMPARs, autoradiography was performed with [^18^F]K-40_OH_. The binding affinity (measured by the dissociation constant) was 19.5 nM, and the binding capacity was 21.9 fmol/mg of tissue (Supplemental Fig. 3). These are within the same range as those of K-2_OH_ (20). In an off-target binding assay, K-40_OH_ showed no marked binding to any major receptors in the central nervous system other than AMPARs (Supplemental Table 1).

### *V*_T_ with pTAC and BP_ND_ Using White Matter as Reference

**FIGURE 4.**
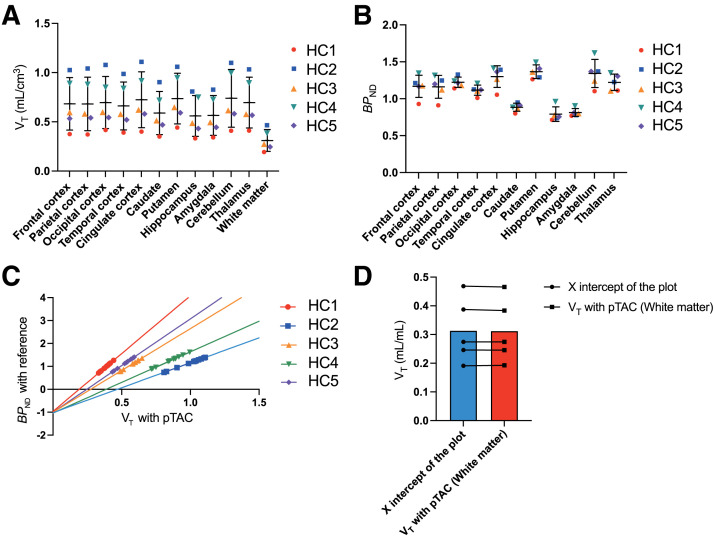
(A) *V*_T_ values of various brain regions computed with pTAC from LGA plots in [^18^F]K-40 PET study. (B) BP_ND_ values of various brain regions calculated with Logan plot analysis using white matter as reference region (without pTAC). (C) Linear regression analysis between *V*_T_ of brain regions (except white matter) computed with pTAC and BP_ND_ using white matter as reference. (D) Comparison of *V*_T_ value, which corresponds to zero point of BP_ND_, using white matter as reference (*x* intercept of plot in (C)) and *V*_T_ of white matter computed with pTAC. *n* = 5. (A and B) Data are shown as mean ± SD. HC = healthy control.

*V*_T_ of various brain regions was calculated with pTACs. *V*_T_ is determined by the ratio of ligand concentration in tissue to the concentration in plasma and is an index representing the extent to which a drug has migrated into the tissue (Fig. 4A) (30). Among the brain regions, we found the smallest *V*_T_ for white matter in [^18^F]K-40. These results suggest that the white matter is applicable as a reference region for K-40 to quantify AMPAR density. White matter is an area in which no AMPARs are present and can be considered a good reference area for AMPAR PET images using [^11^C]K-2 (19,20,22).

Binding potential is a quantitative index of receptor density used in PET receptor imaging and is defined as the ratio of apparent binding capacity and dissociation constant (30). The BP_ND_ value for each tissue was calculated using LGA without pTAC, with the white matter as the reference region (Fig. 4B). We then plotted the relationship between *V*_T_ and BP_ND_ in Figures 4A and 4B, except for white matter (Fig. 4C). *V*_T_ values, which corresponded to the zero point of BP_ND_ when using white matter as a reference (*x* intercept of the plot in Fig. 4C), were almost identical to the *V*_T_ values computed for white matter when using the pTAC (Fig. 4D). These results, in combination with the biochemical study showing no AMPAR expression in white matter (20), suggested that calculating BP_ND_ with LGA using white matter as the reference was feasible for [^18^F]K-40, and the applied algorithm to compute BP_ND_ of LGA is reasonable.

### PET Imaging of [^18^F]K-40 and [^11^C]K-2

**FIGURE 5.**
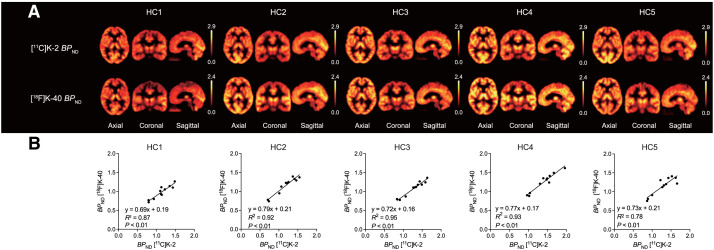
(A) Comparison between [^11^C]K-2 BP_ND_ PET images and [^18^F]K-40 BP_ND_ PET images. (B) Correlation between [^11^C]K-2 and [^18^F]K-40 BP_ND_ values in brain regions in healthy subjects. HC = healthy control.

One subject underwent AMPAR PET imaging with both of [^18^F]K-40 and [^11^C]K-2, and the computed BP_ND_ images of [^18^F]K-40 and [^11^C]K-2 were compared. Figure 5A shows that BP_ND_ images in each case represented similar regional distributions, with a very good correlation between the BP_ND_ values of [^18^F]K-40 and [^11^C]K-2 of each region (Fig. 5B). The linear relationship between BP_ND_ of [^18^F]K-40 and [^11^C]K-2 for 5 subjects on average was *y* = 0.75*x* + 0.18 (*R*^2^ = 0.85). The variation in slope between subjects was sufficiently small, and the *y*-intercept was also sufficiently small compared with the BP_ND_ value. These results suggest that [^18^F]K-40 can be used as an AMPAR PET tracer with a half-life longer than that of [^11^C]K-2.

### Optimization of Time Frame for [^18^F]K-40 PET Imaging

For clinical use, using Bland–Altman plots, we explored the time frame showing the best correlation between BP_ND_ and SUVR.

**FIGURE 6.**
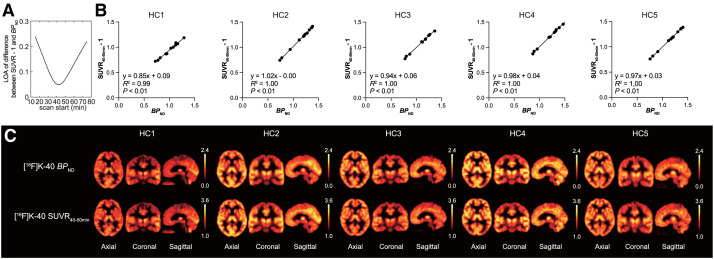
(A) Change in limit of agreement on difference in BP_ND_ and SUVR with varying scan start time wherein frame width is fixed at 10 min. (B) Correlation between BP_ND_ value and SUVR_40–50min_−1 in brain regions in healthy subjects. (C) Comparison between [^18^F]K-40 BP_ND_ PET images and [^18^F]K-40 SUVR PET images at 40–50 min.

The time frame showing the best correlation between BP_ND_ and SUVR, with white matter as a reference, was examined. The tTAC time frame was seamlessly supplemented, and an SUVR-based Bland–Altman analysis was performed by calculating the data accumulated for 10 min from *x* min after administration, with BP_ND_, which used the white matter as the reference region. The difference between SUVR−1 and BP_ND_ was plotted against the start of the scan (Fig. 6A). The smallest difference between SUVR−1 and BP_ND_ was observed approximately 45 min after administration. The limit of agreement, which is defined as a 95% CI of the difference and describes the stability of the difference between BP_ND_ and SUVR−1, is adequately small in the 10-min time frame from 30 to 55 min after administration. On the basis of these results, we found that BP_ND_ and SUVR from 40 to 50 min after administration were highly correlated (Fig. 6B). Averaged across all subjects, the linear relationship between SUVR_40–50min_−1 and BP_ND_ was represented as *y* = 1.00*x* – 0.01 (*R*^2^ = 0.98). Figure 6C shows the BP_ND_ and SUVR images of each subject, and they are highly identical. Thus, the SUVR at 40–50 min is an appropriate surrogate outcome measure for AMPAR density.

## DISCUSSION

[^11^C]K-2 is the world’s first AMPAR PET tracer, and its ^18^F-labeled tracer, K-40, was investigated in humans in this study. We have already confirmed from various angles that [^11^C]K-2 AMPAR PET images reflect the amount of AMPARs in the brain (18,20). K-2 is quickly metabolized to a carboxylic acid form in both the brain and blood. In an early-phase after the intravenous injection of [^11^C]K-2, a substantial amount of K-2 crosses the blood–brain barrier and is quickly metabolized. Only the metabolite K-2_OH_ remains in both the brain and blood and reaches equilibrium. These kinetics, as well as our previous in vitro experiments, proved that [^11^C]K-2 represents cell-surface AMPARs, a physiologically crucial fraction (18,20).

This study revealed that K-40 also has similar characteristics. As obtained in previous studies, the blood concentration of K-2 decreased rapidly after administration and gradually decreased from 10 to 20 kBq/mL (Fig. 2B). Approximately half of K-2 was metabolized about 3 min after administration, and it was almost completely metabolized within 10 min (Fig. 2B). In comparison, K-40 was metabolized slightly faster than K-2, albeit with the same kinetics (Figs. 2A and 2B).

As shown in Figure 3B, K-40, as observed with K-2, showed a good linear relationship with LGA, indicating a reversible equilibrium state. Even when the amount of ligand in the blood was corrected with K-40_OH_ and LGA was performed with the metabolite (K-40_OH_), a good linear relationship was also obtained (Fig. 3B). This indicated that K-40 was quickly metabolized to K-40_OH_ after administration and reached equilibrium with the metabolite. These kinetic characteristics are important features of this PET drug. Previous research has shown that the unchanged K-2 has sufficient blood–brain barrier permeability, but the metabolite K-2_OH_ has slower permeability through the blood–brain barrier and no permeability through cell membranes (18). Therefore, the unchanged K-40 ligand enters the brain immediately after administration and is quickly metabolized, which slows its excretion from the brain and creates an equilibrium state long enough for PET imaging. Furthermore, these results suggest that PET images with the injection of [^18^F]K-40 also represent cell-surface AMPARs, as we observed with [^11^C]K-2. Further in vitro studies are required to confirm this potential property of [^18^F]K-40.

We previously proved that white matter is the appropriate reference region for [^11^C]K-2, since biochemical analysis with resected tissues of patients with refractory epilepsy revealed that there were no AMPAR in the white matter and that the nondisplaceable volume in brain regions other than the white matter was almost equivalent to the *V*_T_ of the white matter (20), which was the smallest in the brain. The same property was demonstrated in the current study, which proved that white matter is also an appropriate reference region for [^18^F]K-40 images. In addition, we found a good correlation between BP_ND_ of [^11^C]K-2 and that of [^18^F]K-40 images, with white matter as a reference (Fig. 5), demonstrating that the [^18^F]K-40 image also represents AMPAR density.

In AMPAR PET imaging using [^18^F]K-40, SUVR using a time frame of 40–50 min after administration provided the best correlation with BP_ND_. The 10-min scan 40 min after administration is useful for clinical practice. The excellent correlation between the SUVR and BP_ND_ constitutes an excellent performance feature of this PET drug. The best correlation between SUVR and BP_ND_ was observed around 40–50 min, although the difference between SUVR and BP_ND_ was within 10% between 25–35 min and 63–73 min (Fig. 6).

Although the AMPAR-specific binding of [^18^F]K-40 is not related to sex in the preclinical study and the results of the present study are applicable to women, further studies to clarify the sexual difference of AMPAR distribution are desirable.

In this study, [^18^F]K-40 and [^11^C]K-2 imaging were performed on the same subjects at intervals of 2–4 wk. For all subjects, highly comparable images were obtained from the 2 PET scans. This indicates the high equivalence of [^18^F]K-40 and [^11^C]K-2, as well as the high reproducibility of AMPAR PET. In the future, test–retest studies will be conducted to further demonstrate the high reproducibility of AMPAR PET.

The small yield of [^18^F]K-40 synthesis (23) is a limitation of this study. This needs to be addressed in future research.

## CONCLUSION

AMPAR PET was launched using an excellent PET drug, [^11^C]K-2. The arrival of the ^18^F-labeled AMPAR PET drug, [^18^F]K-40, further accelerated this trend. [^18^F]K-40 displayed kinetics similar to those of the well-validated [^11^C]K-2, and the BP_ND_ obtained from each showed a very good correlation. The SUVR, using white matter as the reference region, was well proportional to the BP_ND_. Based on the evidence described in this study, [^18^F]K-40 is an excellent AMPAR PET drug for AMPAR quantification without blood sampling.

## DISCLOSURE

Takuya Takahashi and Tomoyuki Miyazaki are the inventors of a patent application for a novel compound that specifically binds to the AMPA receptor (WO 2,017,006,931), including [^11^C]K-2. Takuya Takahashi, Tomoyuki Miyazaki, and Tetsu Arisawa are the founders and also stockholders of Ampametry Co. Ltd., which holds the exclusive license to use [^11^C]K-2. This project was supported by Special Coordination Funds for Promoting Science and Technology under Grant No. 20H05922 and AMED (Grant No. JP24wm0625304 to Takuya Takahashi). This study was also supported by Takeda Science Foundation. No other potential conflict of interest relevant to this article was reported.

## Notes

KEY POINTS **QUESTION:** Does the new ^18^F-labeled PET tracer, [^18^F]K-40, quantify AMPAR density in humans? **PERTINENT FINDINGS:** [^18^F]K-40 showed similar kinetics to the existing AMPAR PET tracer, [^11^C]K-2, with balanced functions of brain uptake, reversible behavior, and AMPAR-specific binding ability. The white matter was applicable as a reference region. The SUVR acquired from a 10-min static scan 40 min after the administration was compatible with BP_ND_. **IMPLICATIONS FOR PATIENT CARE:** [^18^F]K-40 enables off-site production and distribution of the AMPAR PET tracer, accelerating the development of diagnostics for neuropsychiatric disorders.

## References

[1] Malinow R Malenka RC. AMPA receptor trafficking and synaptic plasticity. Annu Rev Neurosci. 2002;25:103–126. 12052905 10.1146/annurev.neuro.25.112701.142758

[2] Kessels HW Malinow R. Synaptic AMPA receptor plasticity and behavior. Neuron. 2009;61:340–350. 19217372 10.1016/j.neuron.2009.01.015 PMC3917551

[3] Zhao Y Chen S Swensen AC Qian WJ Gouaux E. Architecture and subunit arrangement of native AMPA receptors elucidated by cryo-EM. Science. 2019;364:355–362. 30975770 10.1126/science.aaw8250 PMC6701862

[4] Herguedas B Watson JF Ho H Cais O García-Nafría J Greger IH. Architecture of the heteromeric GluA1/2 AMPA receptor in complex with the auxiliary subunit TARP gamma8. Science. 2019;364. 10.1126/science.aav9011 PMC6513756 30872532

[5] Jitsuki S Takemoto K Kawasaki T. Serotonin mediates cross-modal reorganization of cortical circuits. Neuron. 2011;69:780–792. 21338886 10.1016/j.neuron.2011.01.016 PMC3503249

[6] Abe H Jitsuki S Nakajima W. CRMP2-binding compound, edonerpic maleate, accelerates motor function recovery from brain damage. Science. 2018;360:50–57. 29622647 10.1126/science.aao2300

[7] Takahashi T Svoboda K Malinow R. Experience strengthening transmission by driving AMPA receptors into synapses. Science. 2003;299:1585–1588. 12624270 10.1126/science.1079886

[8] Takemoto K Iwanari H Tada H. Optical inactivation of synaptic AMPA receptors erases fear memory. Nat Biotechnol. 2017;35:38–47. 27918547 10.1038/nbt.3710

[9] Aleksandrova LR Phillips AG Wang YT. Antidepressant effects of ketamine and the roles of AMPA glutamate receptors and other mechanisms beyond NMDA receptor antagonism. J Psychiatry Neurosci. 2017;42:222–229. 28234212 10.1503/jpn.160175 PMC5487269

[10] Chang PK-Y Verbich D McKinney RA. AMPA receptors as drug targets in neurological disease: advantages, caveats, and future outlook. Eur J Neurosci. 2012;35:1908–1916. 22708602 10.1111/j.1460-9568.2012.08165.x

[11] Sato M Kawashima Y Goto J. Synthesis and evaluation of novel fluorinated sulotroban-related sulfonamide derivatives as thromboxane A2 receptor antagonists. Eur J Med Chem. 1995;30:403–414.

[12] Fu H Chen Z Josephson L Li Z Liang SH. Positron emission tomography (PET) ligand development for ionotropic glutamate receptors: challenges and opportunities for radiotracer targeting *N*-Methyl-d-aspartate (NMDA), α-amino-3-hydroxy-5-methyl-4-isoxazolepropionic acid (AMPA), and kainate receptors. J Med Chem. 2019;62:403–419. 30110164 10.1021/acs.jmedchem.8b00714 PMC6393217

[13] Arstad E Gitto R Chimirri A. Closing in on the AMPA receptor: synthesis and evaluation of 2-acetyl-1-(4′-chlorophenyl)-6-methoxy-7-[^11^C]methoxy-1,2,3,4-tetrahydroisoquinoline as a potential PET tracer. Bioorg Med Chem. 2006;14:4712–4717. 16621575 10.1016/j.bmc.2006.03.034

[14] Gao M Kong D Clearfield A Zheng Q-H. Synthesis of carbon-11 and fluorine-18 labeled *N*-acetyl-1-aryl-6,7-dimethoxy-1,2,3,4-tetrahydroisoquinoline derivatives as new potential PET AMPA receptor ligands. Bioorg Med Chem Lett. 2006;16:2229–2233. 16455250 10.1016/j.bmcl.2006.01.042

[15] Oi N Tokunaga M Suzuki M. Development of novel PET probes for central 2-amino-3-(3-hydroxy-5-methyl-4-isoxazolyl)propionic acid receptors. J Med Chem. 2015;58:8444–8462. 26469379 10.1021/acs.jmedchem.5b00712

[16] Yuan G Jones GB Vasdev N Liang SH. Radiosynthesis and preliminary PET evaluation of ^18^F-labeled 2-(1-(3-fluorophenyl)-2-oxo-5-(pyrimidin-2-yl)-1,2-dihydropyridin-3-yl)benzonitrile for imaging AMPA receptors. Bioorg Med Chem Lett. 2016;26:4857–4860. 27546294 10.1016/j.bmcl.2016.07.078 PMC5018461

[17] Lee HG Milner PJ Placzek MS Buchwald SL Hooker JM. Virtually instantaneous, room-temperature [^11^C]-cyanation using biaryl phosphine Pd(0) complexes. J Am Chem Soc. 2015;137:648–651. 25565277 10.1021/ja512115s PMC4394387

[18] Arisawa T Miyazaki T Ota W. [^11^C]K-2 image with positron emission tomography represents cell surface AMPA receptors. Neurosci Res. 2021;173:106–113. 34033829 10.1016/j.neures.2021.05.009

[19] Hatano M Miyazaki T Ishiwata Y. Biodistribution and radiation dosimetry of the positron emission tomography probe for AMPA receptor, [^11^C]K-2, in healthy human subjects. Sci Rep. 2021;11:1598. 33452361 10.1038/s41598-021-81002-3 PMC7810729

[20] Miyazaki T Nakajima W Hatano M. Visualization of AMPA receptors in living human brain with positron emission tomography. Nat Med. 2020;26:281–288. 31959988 10.1038/s41591-019-0723-9

[21] Hatano M Nakajima W Tani H. Characterization of patients with major psychiatric disorders with AMPA receptor positron emission tomography. Mol Psychiatry. In press. 10.1038/s41380-024-02785-1 PMC12014498 39406998

[22] Eiro T Miyazaki T Hatano M. Dynamics of AMPA receptors regulate epileptogenesis in patients with epilepsy. Cell Rep Med. 2023;4:101020. 37080205 10.1016/j.xcrm.2023.101020 PMC10213790

[23] Arisawa T Kimura K Miyazaki T. Synthesis of [^18^F] fluorine-labeled K-2 derivatives as radiotracers for AMPA receptors. Nucl Med Biol. 2022;110-111:47–58. 35642985 10.1016/j.nucmedbio.2022.04.009

[24] American Psychiatric Association, DSM-5 Task Force. Diagnostic and Statistical Manual of Mental Disorders: DSM-5. Vol. 5: American Psychiatric Publishing; 2013.

[25] The ICD-10 Classification of Mental and Behavioural Disorders: Clinical Descriptions and Diagnostic Guidelines. World Health Organization. 1992:362.

[26] Diagnostic and Statistical Manual of Mental Disorders, 4th ed. American Psychiatric Publishing; 1994.

[27] Hammers A Allom R Koepp MJ. Three-dimensional maximum probability atlas of the human brain, with particular reference to the temporal lobe. Hum Brain Mapp. 2003;19:224–247. 12874777 10.1002/hbm.10123 PMC6871794

[28] Logan J Fowler JS Volkow ND. Graphical analysis of reversible radioligand binding from time–activity measurements applied to [*N*-^11^C-methyl]-(−)-cocaine PET studies in human subjects. J Cereb Blood Flow Metab. 1990;10:740–747. 2384545 10.1038/jcbfm.1990.127

[29] Logan J. Graphical analysis of PET data applied to reversible and irreversible tracers. Nucl Med Biol. 2000;27:661–670. 11091109 10.1016/s0969-8051(00)00137-2

[30] Innis RB Cunningham VJ Delforge J. Consensus nomenclature for in vivo imaging of reversibly binding radioligands. J Cereb Blood Flow Metab. 2007;27:1533–1539. 17519979 10.1038/sj.jcbfm.9600493

[31] Logan J Fowler JS Volkow ND Wang GJ Ding YS Alexoff DL. Distribution volume ratios without blood sampling from graphical analysis of PET data. J Cereb Blood Flow Metab. 1996;16:834–840. 8784228 10.1097/00004647-199609000-00008

